# Spectral peak analysis and intrinsic neural timescales as markers for the state of consciousness

**DOI:** 10.1016/j.nicl.2024.103698

**Published:** 2024-10-30

**Authors:** Ezequiel Pablo Espinosa, Di Zang, Andrea Buccellato, Zengxin Qi, Xuehai Wu, Samira Abbasi, Yasir Catal, Stephan Lechner, Federico Zilio, Georg Northoff

**Affiliations:** a Department of Internal Medicine, University of Genoa, 16132 Genoa, Italy; b Department of Neurosurgery, Huashan Hospital, Shanghai Medical College, Fudan University, Shanghai, China; c National Center for Neurological Disorders, Shanghai, China; d Shanghai Key Laboratory of Brain Function and Restoration and Neural Regeneration, Shanghai, China; e State Key Laboratory of Medical Neurobiology and MOE Frontiers Center for Brain Science, School of Basic Medical Sciences and Institutes of Brain Science, Fudan University, China; f Department of Neurosurgery, China-Japan Friendship Hospital, Beijing, China; g University of Ottawa, The Royal’s Institute of Mental Health Research, Brain and Mind Research Institute, Ottawa K1Z 7K4 ON, Canada; h Padua Neuroscience Center, University of Padova, 35131, Padova, Italy; i Department of Biomedical Engineering, Hamedan University of Technology, Hamedan 65169-13733, Iran; j Research Group Neuroinformatics, Faculty of Computer Science, University of Vienna, 1010 Vienna, Austria; k Vienna Doctoral School Cognition, Behavior and Neuroscience, University of Vienna, 1030 Vienna, Austria; l Department of Philosophy, Sociology, Education and Applied Psychology, University of Padova, 35139, Padua, Italy

**Keywords:** Diagnosis, Disorders of consciousness, EEG, Alpha peak, Intrinsic neural timescales

## Abstract

Resting state EEG in patients with disorders of consciousness (DOC) is characterized by an increase of power in the delta frequency band and a concurrent decrease in the alpha range, equivalent to a weakening or disappearance of the alpha peak. Prolongation of Intrinsic Neural Timescales (INTs) is also associated with DOCs. Together, this raises the question whether the decreased alpha peak relates to the prolonged INTs and, importantly, how that can be used for diagnosing the state of consciousness in DOC individuals. Analyzing resting state EEG recordings from both healthy subjects and DOC patients, we measure INTs through autocorrelation window (ACW) and utilize peak analysis to quantify the weakening of the alpha peak. First, we replicate previous findings of prolonged ACW in DOC patients. We then identify significantly lower alpha peak measures in DOC compared to controls. Interestingly, spectral peaks shift from the alpha to the theta range in several DOC subjects while such change is absent in healthy controls. Next, our study reveals a close relationship between ACW and alpha peak in both healthy and DOC subjects, a correlation that holds for theta peaks in DOC. Further, the prolonged ACW correlates with the state of consciousness, as quantified by the Coma Recovery Scale-Revised (CRS-R), and mediates the relationship between theta peak and CRS-R. Finally, through split analyses and machine learning, we show that ACW and alpha peak measures conjointly distinguish healthy controls and DOC patients with high accuracy (95.5%). In conclusion, we demonstrate that the prolongation of ACW, together with spectral peak measures, holds promise to serve as additional EEG biomarkers for diagnosing the state of consciousness in DOC subjects.

## Highlights

• Intrinsic Neural Timescales (INTs) are prolonged in patients with disorders of consciousness (DOC) compared to controls.
• Alpha frequency power peaks are prominent in healthy EEGs but uncommon and diminished in DOC patients.
• Peak analysis quantifies the reduction in alpha peaks in DOC patients relative to controls.
• Prolonged INTs and reduced alpha peaks may serve as biomarkers for consciousness assessment.

## 1 Introduction

The diagnosis of disorders of consciousness (DOC) remains an ongoing challenge for both clinicians and researchers alike, particularly regarding differential diagnosis between minimally conscious state (MCS) and unresponsive wakefulness state (UWS), also known as vegetative state (VS). In recent decades, neurobehavioral assessment tools such as the Coma Recovery Scale–Revised (CRS-R) (Giacino et al., 2014, Giacino et al., 2004) have represented a significant improvement over diagnosis by clinical consensus (Wang et al., 2020) allowing for quantitative and standardized diagnosis. Extending such behavioral assessment, recent research has focused on identifying more objective and reproducible neurobiological markers through the use of neuroimaging.

Among these approaches, EEG has gained attention due to its economic feasibility and potential for bedside use. Several EEG markers, such as the perturbational complexity index (PCI) and other measures of informational complexity (Bodart et al., 2017, Casali et al., 2013, Liu et al., 2023b, Sinitsyn et al., 2020, Sitt et al., 2014, Zilio et al., 2023), evoked potentials (Hermann et al., 2021, Pruvost-Robieux et al., 2022) and spectral analysis (Chennu et al., 2014, Colombo et al., 2023, Goldfine et al., 2011, Lechinger et al., 2013) have shown promising results. However, clinical usage of these markers for differential diagnosis remains an ongoing challenge.

Research on spectral analysis has revealed that the alpha frequency range (7.5 – 13 Hz) exhibits a decrease in power in DOC patients (Engemann et al., 2018, Lechinger et al., 2013, Rossi Sebastiano et al., 2015, Sitt et al., 2014). Recent machine learning studies have further confirmed the importance of alpha, as well as the theta and delta bands, as key electrophysiological markers in classifying DOC patients and discriminating between MCS and UWS/VS (Engemann et al., 2018, Sitt et al., 2014). Typically, the alpha band exhibits a peak in power among healthy subjects (Klimesch, 1999, Klimesch, 1997). However, the exact quantification of the alpha peak including its decrease in power in DOC individuals and its potential relation to the state of consciousness remains an open issue. Addressing this gap in our knowledge constitutes the primary goal of our paper.

Extending beyond spectral measures, recent studies have focused on Intrinsic Neural Timescales (INTs), the timescales at which single neurons and/or neural populations process incoming information (Hasson et al., 2008, Murray et al., 2014, Wasmuht et al., 2018). Research on INTs has revealed that the brain exhibits a temporal hierarchical organization: unimodal regions accumulate and process information over shorter timescales, while transmodal regions integrate and process stimuli across longer time durations (Golesorkhi et al., 2021a). INTs have recently been posited as a key mechanism for consciousness in the Temporo-spatial Theory of Consciousness (TTC) (Northoff and Zilio, 2022, Zilio et al., 2023, Zilio et al., 2021), according to which they allow the brain to encode contents within their respective contexts. In this framework, TTC posits INTs as giving rise to the structure of consciousness, representing the background in the figure – background dyad.

Narrowing our focus on DOCs, INTs, measured through the autocorrelation window (ACW), are abnormally prolonged in states of decreased consciousness, such as sleep, anesthesia, and DOC (Buccellato et al., 2023, Zilio et al., 2021). This prompts the question of whether the prolongation of ACW in DOC patients could relate to the weakening or disappearance of their alpha peak, and whether the two conjointly modulate the state of consciousness.

The primary goal of our study was to investigate and quantify the changes in the alpha peak as well as how they relate to INTs, measured through ACW, in both healthy controls and DOC patients. We analyzed 25 EEG recordings obtained from 25 healthy controls and 95 EEG recordings obtained from 88 DOC patients. To quantify the weakening or disappearance of the alpha peak in DOC we utilized peak analysis, identifying five peak-related measures – *power*, *power ratio*, *prominence*, *width* and *frequency*. To avoid restricting our analysis to the alpha range, we also incorporated two non-peak measures, *maximum power* and *minimum mower*. These measures explore the delta and gamma frequency ranges, respectively, taking into account the inverse exponential trend of the PSD. For clarity, we refer to the peak-related measures as “peak measures” while both peak-related and non-peak-related measures combined as “spectral measures”. Additionally, we aimed at replicating previous results of a prolonged ACW in DOC (Buccellato et al., 2023, Zilio et al., 2021). Given previous findings, we hypothesized that DOC subjects would show a prolongation of ACW alongside a significant reduction in our alpha peak measures. The second specific aim involved establishing a relationship between alpha peak measures, ACW and the state of consciousness, as measured by CRS-R. We anticipated negative correlations between alpha peak measures and ACW, both of which are expected to relate to the level of consciousness (CRS-R). The third specific aim probed whether our measures enable the prediction of the state of consciousness, as tested through split analyses and machine learning. Building on our previous work (Buccellato et al., 2023, Zilio et al., 2021), we hypothesized that ACW, particularly in its relation to alpha peak measures, might carry potential for accurately classifying patients based on the state of consciousness, as measured by CRS-R.

## 2 Methods and Materials

### 2.1 Participants

95 resting state EEG recordings (MCS = 47; UWS = 48) were obtained from 88 patients with disorders of consciousness (mean age = 46.91 ± 15.82; sex-ratio = 2.38; etiology: stroke = 44; anoxia = 6; TBI = 38) from July 2016 to June 2019. The difference between the number of EEG recordings and the number of patients is explained by seven patients drifting from one diagnostic category to another during follow-up (i.e. from UWS to MCS or vice versa).

On admission patients were assessed by trained clinicians with the Glasgow Coma Scale (GCS) (Teasdale and Jennett, 1974). Further evaluation was obtained with the JFK Coma Recovery Scale–Revised (CRS-R) (Giacino et al., 2004) immediately preceding the recording session. The recording session lasted for a minimum of 5 min and employed a 256-channel system (GES 300, Electrical Geodesics, Inc., USA). Before starting the recording, examiners performed the Arousal Facilitation Protocol (Giacino et al., 2004) to induce wakefulness. No sedative agents were administered 24 h prior to recording. Possible sources of electronic noise were reduced, and participants wore soundproof earmuffs (3 M Company) to attenuate environmental noise. Given their reduced state of consciousness, patients were not able to follow commands. For this reason, whether patients maintained their eyes opened or closed during the recording session, or whether they showed spontaneous movements was not under the examiner’s control and not tracked.

For the control sample, 25 healthy participants (age 24.56 ± 0.71 years; M/F sex-ratio = 0.94) underwent a similar procedure: a 5-minute resting-state recording session utilizing the same EEG recording system (GES 300, Electrical Geodesics, Inc., USA). Controls were asked to lay in bed and keep their eyes open: this was done to mimic the recording experience of DOC patients as much as possible. EEG data was re-referenced online to Cz and acquired at a sampling rate of 1000 Hz, while keeping impedance of all electrodes below 20 KΩ.

### 2.2 Ethics statement

Before participation, informed written consent was obtained from all participants (or from their caregivers). The study was approved by the Ethical Committee of the Huashan Hospital of Fudan University (approval number HIRB-2014–281) and conducted in accordance with the Declaration of Helsinki guidelines.

### 2.3 Pre-processing

First, data was down sampled to 250 Hz. Then, a band-pass finite impulse response (FIR) filter between 0.5 and 40 Hz (Hamming window) was applied. Noisy channels were identified and excluded from further analysis through a semiautomatic procedure.

Criteria for rejection of noisy channels were the following: flatline channels (channels showing no activity for more than 5 s), highly correlating channels (threshold set at 0.8), low-frequency drifts, noisy channels and short-timed bursts not related to neural activity (threshold at SD = 5 for data portions relative to baseline). Removed channels were then interpolated with a spherical method and channel activity was re-referenced to the common average reference. Finally, all recordings were clipped to a length of exactly 5 min. Artifacts (i.e. eye movements, muscular noise, and heart activity) were removed through independent component analysis (ICA) (Delorme and Makeig, 2004).

### 2.4 Electrode selection

For our research we focused on three frontal electrodes (i.e. Fz, F1, F2) and three occipital electrodes (i.e. Oz, O1, O2), selected based on the key role played by these regions in DOC, as evidenced by consistent differences between MCS and UWS/VS (Yesong Liu et al., 2023). Specifically, alpha band power in frontal and in occipital regions has proven to be higher in MCS compared to UWS/VS (Naro et al., 2018, Piarulli et al., 2016, Rossi Sebastiano et al., 2015), while delta power was found to be higher in UWS/VS over the same areas (Naro et al., 2018, Piarulli et al., 2016, Rossi Sebastiano et al., 2015). Therefore, both spectral and ACW measurements were calculated separately for these six electrodes and averaged among the frontal and occipital electrodes, respectively.

### 2.5 Spectral analysis

The Power Spectral Density (PSD) describes the power for each frequency component of a signal. To estimate the PSD, the Welch method was used. The method splits the EEG timeseries into windows (3 s, for our study) with a certain degree of overlap between them (50 %, for our study) and computes a Fast Fourier Transform (FFT) for each window. It then calculates the absolute value of Fourier coefficients for each frequency, and a Hamming window is applied to all segments. Finally, the PSD is estimated by averaging across all individual periodograms.

This procedure was performed for the selected electrodes (i.e. three frontal: Fz, F1, F2; three occipital: Oz, O1, O2), and PSDs were averaged across frontal and occipital electrodes for each subject, obtaining two PSDs: one frontal and one occipital. The Y-axis was set to logarithmic scale (base 10), and further analysis was performed on such values.

### 2.6 Peak analysis

For peak analysis, the MATLAB function “findpeaks” (Signal Processing Toolbox) was used. The function identifies local maxima and returns its X and Y coordinates, as well as the peak’s *width* and *prominence*. This function has found widespread use, with studies ranging from X-ray photoelectron spectroscopy (Lubenchenko et al., 2020), to gait analysis in patients with Parkinson’s disease (Pham et al., 2017) and has already been applied to EEG spectral analysis (Rodriguez-Larios and Alaerts, 2021, Scally et al., 2018).

Peak and non-peak measures are defined as follows:

‘*Power’*: the local maxima itself (i.e. Y-coordinate). *Power* is therefore the ‘tip’ of the peak (i.e. the highest point): the word ‘peak’ should then be intended as representing the region of the PSD encompassing a *power*, a *width*, a *prominence*, and a *frequency*.

‘*Frequency’*: the corresponding X-coordinate of the local maxima (i.e. the Power). We briefly acknowledge the difference between alpha peak frequency (APF) and our “*frequency*”. Some studies (Buccellato et al., 2023) have utilized frequency sliding (Cohen, 2014) to measure APF. This method significantly differs from ours and results in a time series of instantaneous frequencies. In contrast, our usage of the term “*frequency*” refers to and quantifies the frequency at which the alpha peak reaches its highest power.

‘*Prominence’*: *prominence* (or ‘relative height’) is defined as the difference between the height of the peak and its highest minimum. To visualize this measure, the following example is used, taken from the MATLAB documentation. Picture tracing a horizontal line through the highest point of the peak, to the left and to the right of it, until the line either reaches the endpoint of the signal or crosses the signal at the slope of a higher peak. Then, calculate the minimum point in these two segments (to the left and to the right of the peak). The greater between the two values is the highest minimum and is used for the calculation of the *prominence*.

‘*Width’*: *width* is computed as the distance between the peak’s left and right points where its descending slopes intersect with a horizontal reference line. This reference line is set, by default, to be at a height equal to the middle point of the *prominence*.

‘*Maximum Power’*: the maximum power value in the PSD.

‘*Minimum Power’*: the minimum power value in the PSD.

‘*Power Ratio’*: the ratio between *power* and *maximum power*.

For simplicity, we will refer to all seven measures as “spectral measures” and to the peak-related measures as “peak measures”.

### 2.7 Modulating peak analysis

To modulate the analysis, the “findpeaks” function provides the option to apply various thresholds to exclude undesired peaks from the output. One such threshold is the ‘minimum peak width’, which can be set to exclude peaks with widths below the specified threshold from the function’s output. The only thresholds used in our analysis were: minimum distance between peaks (set to 0.4 in our study) and minimum peak prominence (set to 0.1 in our study). The values just noted, and the values obtained for the spectral measures previously described, are expressed as units of X and Y coordinates, respectively. As an example, a peak whose *width* ranges from *x*_1_ = 9 to *x*_2_ = 11 will have a *width* of |*x*_2_| – |*x*_1_|= 2; a peak whose *power* is *y*_1_ = −3.2 and highest minimum is *y*_2_ = −3.7 (in logarithmic scale) will have a *prominence* of |*y*_2_| – |*y*_1_| = 0.5. Units for the X-axis measures (i.e. *width* and *frequency*) are therefore expressed in Hz, whereas units for the Y-axis measures (*power*, *prominence*, *maximum mower*, and m*inimum power*) are in the unit of power.

We additionally note that the analysis was performed on the PSD with power set to logarithmic scale in base 10, a commonly used way to visualize power spectral graphs. The function returns the PSD graph with the identified peaks annotated, and all graphs (both frontal and occipital) were visually inspected.

**Fig. 1:**
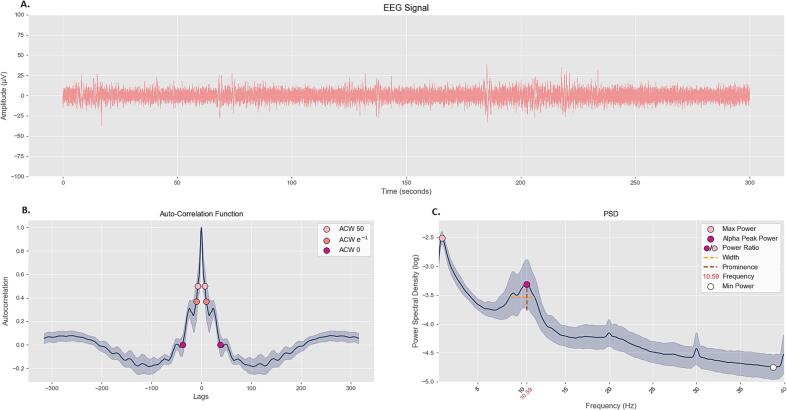
**From EEG to Auto-Correlation Function and Power Spectral Density.** Starting from an EEG signal (**A**) we measure Intrinsic Neural Timescales (INTs) using the Autocorrelation Function (**B**) and obtain the Power Spectral Density (PSD) via a Fast Fourier Transform (**C**). Panel (**C**) illustrates the measures we acquired through peak analysis.

A ‘theta peak’ was defined as a peak having its *frequency* in the 3–7.5 Hz range; an ‘alpha peak’ was defined as a peak having its *frequency* in the 7.5–13 Hz range. When multiple peaks were found, the peak with greater *prominence* was selected and subsequently used for analysis. If the function could not identify any peaks, then the PSD was counted as ‘no peak’ (or ‘flat’). Peak and non-peak measures can be visualized in Fig. 1C.

### 2.8 Intrinsic neural timescales – ACW-50, ACW-e^−1^, ACW-0

The Auto-Correlation Function (ACF) estimates the correlation between a signal and a copy of itself, delayed in time. The autocorrelation window (ACW) marks the first lag of ACF where the value of autocorrelation reaches a desired threshold. These values serve as an estimation of how quickly a signal decorrelates with itself.

For ACW-50, ACW e^−1^, and ACW-0, time lag thresholds were set, respectively, at 0.5, e^−1^ and 0. Determining the appropriate time lag for autocorrelation of EEG timeseries is a complex task. Previous studies have utilized both ACW-50 (Golesorkhi et al., 2021a, Zilio et al., 2021, Zilio et al., 2023) and the more novel ACW-0 (Buccellato et al., 2023, Golesorkhi et al., 2021a). For our study, we included these measures and added ACW-e^−1^, which has been proposed in the past (Bassingthwaighte et al., 1994).

Calculations were computed for the selected electrodes (i.e. three frontal: Fz, F1, F2; three occipital: Oz, O1, O2) and averaged for frontal and occipital electrodes respectively, obtaining one value for frontal electrodes, and one value for occipital electrodes, for each of the three ACW thresholds, respectively.

### 2.9 Statistical analysis

For all samples, before proceeding with further analysis, a Shapiro-Wilk test for normality was performed: for differences between spectral measures in controls and DOC patients parametric (independent *t* test) and non-parametric (Mann-Whitney *U* test) statistical tests were performed accordingly.

For correlations between spectral measures and ACW, no specific assumptions of linear or non-linear relationships between the variables were made. Therefore, Pearson or Spearman correlation coefficients were estimated according to the normality of sample distributions. An identical procedure was followed for figures in Supplementary material. Significance level was set to 0.05 and p values were adjusted for multiple comparisons with the Benjamini-Hochberg correction where appropriate.

For all correlations involving CRS-R, given the variable’s ordinal nature, only Spearman’s coefficient was calculated. Mediation analysis was performed with a bootstrap method (n = 5000) after standard scaling. Importantly, the exogenous variable CRS-R was treated as ordinal.

For differences in the rates of presence or absence of peak a Chi-Squared test was used. For the median split results an independent *T*-test (for *power ratio* and *power*) and a Mann-Whitney *U* test (CRS-R) were utilized.

It is essential to highlight that the frontal and occipital groups did not always consist of identical subjects. In some instances, a subject exhibited a peak in the frontal region without a corresponding peak in the occipital region, and vice versa. The reader should infer that frontal and occipital groups comprise distinct sets of subjects, unless expressly indicated otherwise.

### 2.10 Machine learning

For the machine learning analysis, we employed a Support Vector Machine (SVM), due to its robustness in high-dimensional data and its effectiveness in dealing with non-linear decision boundaries (Rathgamage Don, 2018). The analysis used the Matlab ‘fitcecoc’ function, which implements Error-Correcting Output Codes (ECOC) for multiclass classification (Escalera et al., 2010, Escalera et al., 2009). The function trains a classifier using SVMs with a one-against-one (OvO) coding design. The OvO approach constructs N×(N − 1)/2 binary SVM models, where N represents the number of classes in the dataset, ensuring effective classification across multiple classes (Escalera et al., 2010). The SVM model was trained on the training dataset along with its corresponding class labels. Throughout the training process, the model optimized the hyperparameters and determined the optimal decision boundary to maximize classification accuracy.

To evaluate the generalization performance of the SVM model and mitigate overfitting, we employed 10-fold cross-validation.

The performance of the trained SVM model was assessed using accuracy, precision, recall, F1-score, and confusion matrix analysis. Additionally, receiver operating characteristic (ROC) curves and area under the curve (AUC) values were computed to assess the model’s discriminative ability across different thresholds. The average performance across all folds was then calculated to provide a robust estimate of the model’s effectiveness in classifying unseen data.

### 2.11 Software

Pre-processing, ICA, peak analysis, spectral analysis, ACW extraction and machine learning were performed on the MATLAB software (The MathWorks, 2023b) and the EEGLAB toolbox. Statistical tests were performed on Python (3.11.5), using the SciPy package, except the mediation model, which was carried out on R (4.1.3) using the lavaan package (Rosseel, 2012).

## 3 Results

### 3.1 Alpha and theta peaks in controls and DOC

The occurrence of a power peak in the alpha frequency range (7.5–13 Hz) is a common observation in the PSD of healthy subjects (Başar, 2012). We started by determining whether we could replicate this finding in our control group (n = 25). Most subjects (frontal = 22; occipital = 22) exhibited a distinct peak in the frequency range between 7.5 and 13 Hz, with only 3 frontal channels subjects and 3 different occipital channels subjects not displaying any kind of peak.

Conversely, DOC patients show a decrease in alpha power and a shift to lower frequency bands, such as theta and delta (Lechinger et al., 2013). We examined whether this pattern was reflected in our dataset and reproducible with our methodology. A Chi-Squared test compared controls and DOC patients on the observed occurrences of alpha peaks, theta peaks and no peaks (Table 1). Results proved to be statistically significant for both frontal, X^2^ (2, N = 120) = 70.42, p < 0.001, and occipital electrodes, X^2^ (2, N = 120) = 53.21, p < 0.001. Alpha peaks were significantly more prevalent in the control group compared to the DOC, which, in contrast, showed a higher incidence of theta peaks and ‘flat’ PSDs.

**Table 1:** Alpha, theta and no peaks in controls and DOC subjects.

	**N° of Recordings**	**Alpha Peak**	**Theta Peak**	**No Peak**
**Controls (frontal)**	25.	22 (88 %)	0	3 (12 %)
**MCS (frontal)**	48	3 (6.3 %)	11 (22.9 %)	34 (70.8 %)
**UWS (frontal)**	47	4 (8.5 %)	12 (25.5 %)	31 (66 %)
**MCS + UWS (frontal)**	95	7 (7.4 %)	23 (24.2 %)	65 (68.4 %)
**Controls (occipital)**	25.	22 (88 %)	0	3 (12 %)
**MCS (occipital)**	48	8 (16.7 %)	11 (23 %)	29 (60.4 %)
**UWS (occipital)**	47	5 (10.6 %)	16 (34 %)	26 (55.3 %)
**MCS + UWS (occipital)**	95	13 (13.7 %)	27 (28.4 %)	55 (57.9 %)

MCS: minimally conscious state; UWS: unresponsive wakefulness state.

We then sought to determine whether our alpha peak measures would exhibit differences between controls and the few DOC patients who showed alpha peaks. We conducted Mann-Whitney *U* and independent t tests on 22 controls and 7 DOC for frontal electrodes, and 22 controls and 13 DOC for occipital electrodes. Overall, we found strong differences across six of our seven measures. Specifically, controls displayed higher *power*, *power ratio*, *frequency*, *prominence* and *minimum power*, while *maximum power* was higher in DOC, and *width* showed no statistically significant differences among the two groups. Results are summarized in Fig. 3.

**Fig. 2:**
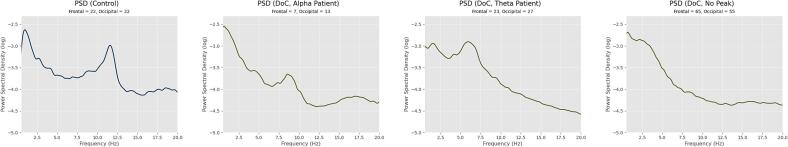
**Variations in Power Spectral Density.** Four distinct PSDs are displayed: a clear peak in the alpha range is visible for the healthy control (utmost left plot), which sees a considerable decrease in *power*, *prominence* and *frequency* in DOC patients (middle left plot), shifting to the theta range (middle right) or disappearing entirely (utmost right plot).

**Fig. 3:**
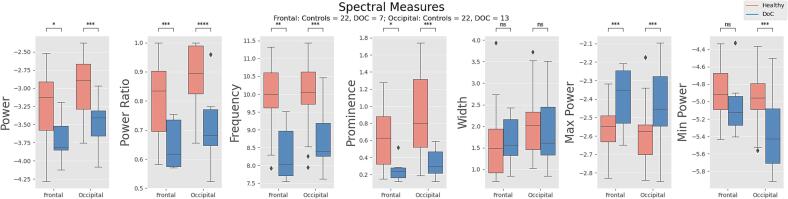
**Alpha spectral measures in controls and DOC patients.** Through peak analysis we identify seven spectral measures, five directly related to the alpha peak and two non-peak related. Controls exhibit higher values across almost all measures, except for *width* (showing no statistically significant differences) and *maximum power* (higher in DOC patients). Ns: p ≥ 0.05; *0.01 < p < 0.05; ^**^0.001 < p < 0.01; ***0.0001 < p < 0.001; ****p ≤ 0.0001.

### 3.2 Autocorrelation window (ACW) in controls and DOC

**Fig. 4:**
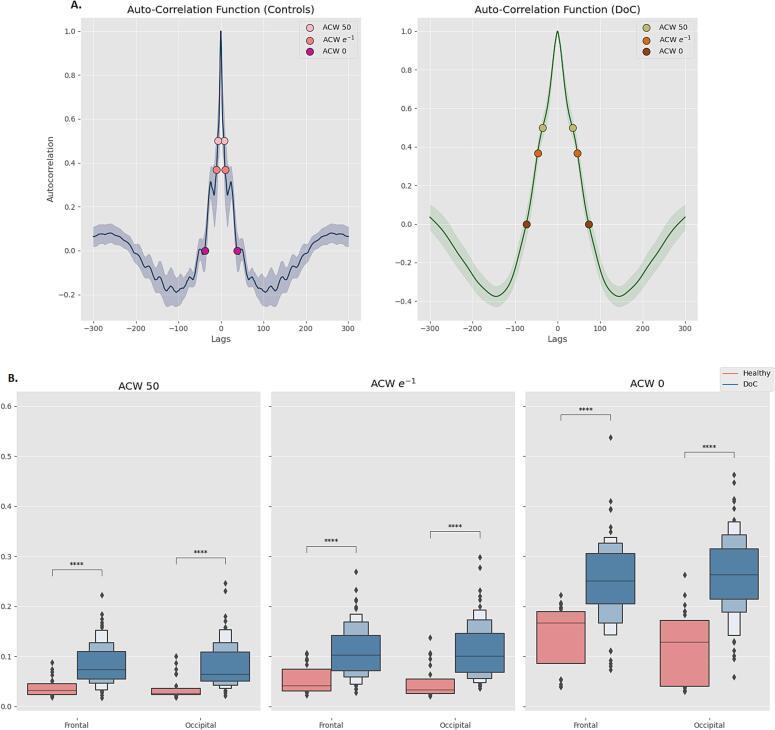
**ACW, controls and DOC.** Patients with Disorders of Consciousness exhibit prolonged ACW: this difference is clearly visible when comparing two Autocorrelation Functions side by side (**A**). In our study we replicate this finding and extend it to ACW-e^−1^ (**B**). *0.01 < p < 0.05; ^**^0.001 < p < 0.01; ***0.0001 < p < 0.001; ****p ≤ 0.0001.

ACW has been previously demonstrated to be prolonged in disorders of consciousness (Buccellato et al., 2023, Zilio et al., 2021). In this study, we replicate this finding by comparing the entire control group (n = 25) with the entire DOC group (n = 95) and extend it to different ACW measures (i.e. ACW-0, ACW e^−1^ and ACW-50). Mann-Whitney *U* tests demonstrated high statistical significance (p < 0.001). Fig. 4b displays the results.

### 3.3 Relationship between INTs and alpha peak

Given the observed differences of both INTs and alpha peaks in our DOC subjects, we focused on the potential link between the two.

#### 3.3.1 Controls

We performed Pearson’s and Spearman’s correlations between ACW and spectral measures. Controls (frontal = 22, occipital = 22) displayed strong negative correlations for *power* and *power ratio*, while *maximum power* showed very strong positive correlations with ACW, reaching high levels of statistical significance. Among the other measures, *prominence*, *width* and *minimum power* showed weak to moderate negative correlations, without reaching statistical significance. For *frequency*, correlation coefficients were positive, but not significant.

#### 3.3.2 DOC

We then conducted the same analysis on DOC subjects who displayed alpha peaks (frontal = 7; occipital = 13) and, afterwards, on those with a theta peak (frontal = 23; occipital = 27).

Overall, results appeared similar to the ones for controls, displaying moderate to strong negative correlations for *power*, *power ratio*, and very strong positive correlations for *maximum power*. Contrary to controls, correlation coefficients for *prominence* and *width* were positive. *Frequency* displayed weak to moderate correlations (e.g. r = 0.58 for ACW-0, in frontal electrodes), but none having *p* value < 0.05.

When shifting our focus to DOC patients displaying theta peaks (frontal = 23, occipital = 27), results closely resembled those described for alpha, revealing strong and very strong negative correlations for *power* and *power ratio*, and positive correlations for *maximum power*, with *prominence*, *width* and *frequency* failing to reach statistical significance (Supplementary Fig. 1). Interestingly, correlation coefficients for *prominence* and *width* were negative, similar to controls but in contrast to alpha peak DOC subjects.

**Fig. 5:**
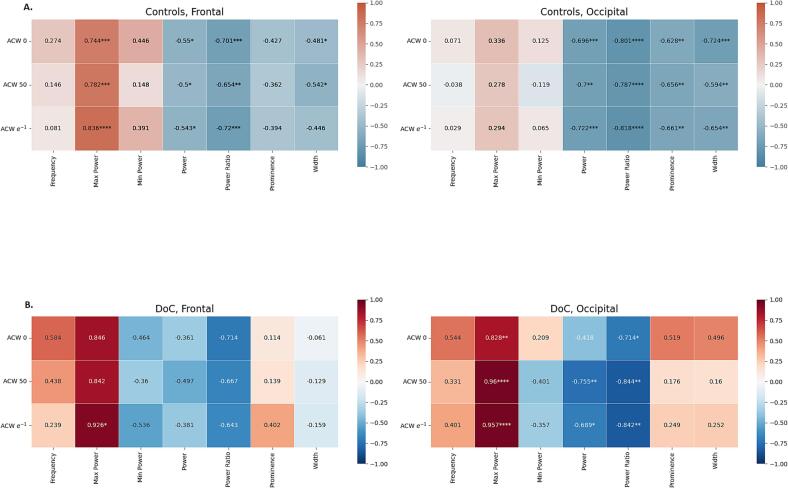
**Correlations between ACW and alpha spectral measures in controls and DOC.** ACW and alpha peak hold a strong relationship, especially for *power*, *power patio* and *maximum power*, which exhibit very strong correlations. These correlations are present both in healthy controls (**A**) and in DOC individuals (**B**). *0.01 < p < 0.05; **0.001 < p < 0.01; ***p < 0.001.

In summary, our results reveal strong negative correlations with ACW for *power* and *power ratio* and nearly perfect positive correlations for *maximum power*. *Prominence*, *width*, *frequency* and *minimum power* displayed weak to moderate correlations but did not reach significance level. Full results are presented in Fig. 5.

### 3.4 INTs as mediator between theta peak and CRS-R

**Fig. 6:**
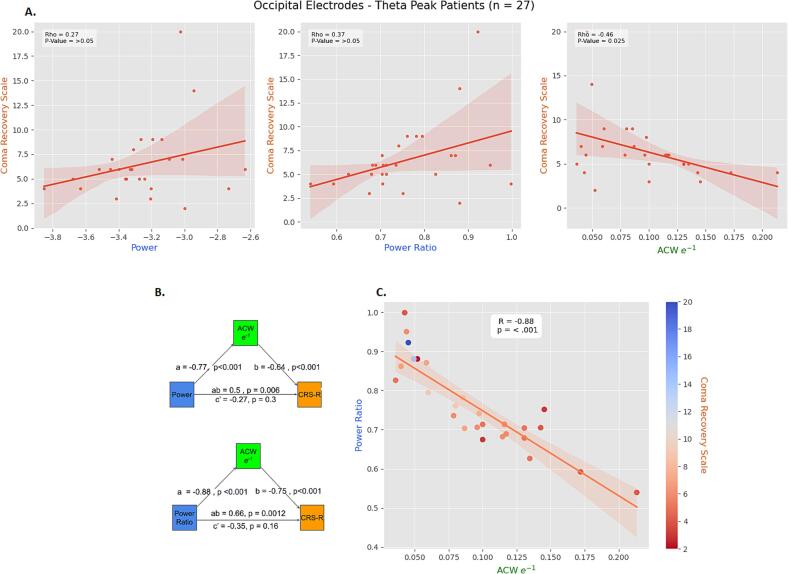
**Mediation analysis.** Our mediation analysis starts from observing strong correlations between ACW and peak measures (Fig. 5) and correlations between ACW and CRS-R, which in turn, are absent between peak and CRS-R (**A**). The mediation models (**B**) hint at ACW possibly serving as mediators between peak and CRS-R. Panel (**C**) displays the strong correlation between power ratio and ACW, with CRS-R showing a weak tendency to decrease as ACW increases.

Next, our inquiry turned to the possible relationship between INTs and the state of consciousness as measured by the Coma Recovery Scale-Revised (CRS-R). Two significant negative correlations were found between ACW and CRS-R in DOC patients displaying theta peaks (frontal = 23, occipital = 27): ACW-e^−1^ (r = −0.46, p = 0.025) and ACW-0 (r = −0.46, p = 0.025) for occipital electrodes. No significant correlations were found in alpha peak DOC patients (frontal = 7, occipital = 13) or across the entire DOC group (n = 95), as well as between spectral measures and CRS-R (Fig. 6A).

Considering the above findings, we hypothesized a mediation between the three elements: ACW could serve as a mediator, peak measurements acting as independent variables and CRS-R as the outcome. Given the low sample size of alpha peak patients, mediation models were attempted only for DOC subjects who displayed theta peaks. The variables used for the models were selected based on the correlational findings described above: ACW-e^−1^ and ACW-50 had demonstrated significant correlations with CRS-R, while *power*, *power ratio* and *maximum power* had shown the strongest links to ACW. Two significant models were identified for occipital electrodes (n = 27), while no such results were found for frontal electrodes (n = 23). It is essential to approach these mediation results with caution due to the cross-sectional nature of the data and the low sample size (Fairchild and McDaniel, 2017).

Fig. 6b summarizes the results. The predictor variable for the first model was *power*, while ACW-e^−1^ served as the mediator. This model revealed an indirect effect of *power* on CRS-R (ab = 0.5, p = 0.006). The second model, using *power ratio* as the independent variable and ACW-e^−1^ as the mediator, also demonstrated an indirect effect of ab = 0.66, p = 0.0012.

Taken together, our findings indicate negative correlations between ACW and CRS-R in patients displaying theta peaks. Additionally, we identified two significant mediation models for theta peak subjects, where *power* or *power ratio* served as independent variables, ACW-e^−1^ acted as mediator and CRS-R as the outcome.

### 3.5 Sorting according to the state of consciousness

Next, we aimed at classifying individual DOC patients into groups based on spectral measures and INTs. Initially, we sought to determine whether DOC patients with a spectral peak (frontal = 30; occipital = 40) exhibited differences in ACW compared to those without peaks (frontal = 65; occipital = 55). We categorized patients into two groups based on peak presence or absence and employed Mann Whitney *U* and independent t tests. Significant differences were identified for all three ACW measures, albeit exclusively in occipital electrodes. On average, patients with a theta peak displayed shorter ACW values, with the most significant difference being ACW-50 in occipital electrodes: median ACW-50 values in subjects with a peak and without a peak were 0.083 s and 0.059 s, respectively, (Mann-Whitney *U* = 743, p = 0.022; see Supplementary Fig. 2).

**Fig. 7:**
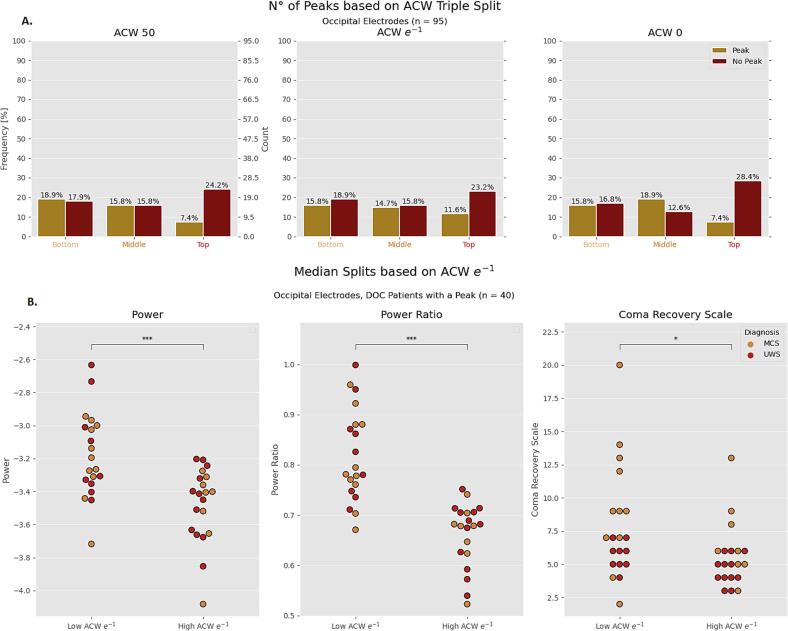
**ACW splits.** The link between ACW and spectral peaks in DOC subjects extends from the weakening to the disappearance of all spectral peaks: patients in the top quantile for ACW length possess, on average, a lower chance of displaying a spectral peak (**A**). ACW allows segregation of peak measures and CRS-R (**B**). Ns: p ≥ 0.05; *0.01 < p < 0.05; **0.001 < p < 0.01; ***0.0001 < p < 0.001; ****p ≤ 0.0001.

Next, we split DOC subjects (n = 95) into three equal parts based on their ACW, identifying a group with low ACW, a group with intermediate ACW and a group with high ACW (see Fig. 7A). We then counted the presence and absence of peaks in each group. On average, patients with a longer ACW (i.e. ACWs in the top quantile) had fewer peaks (alpha or theta) across all ACW measures, except for ACW-0 in frontal electrodes, where the peak count was lowest for the middle quantile and intermediate for the top quantile. A Chi Squared test yielded only one significant result: ACW-0 in the occipital group, X^2^ (2, N = 95) = 10.9, p = 0.013.

We additionally performed a median split analysis, which included DOC patients exhibiting a peak (alpha or theta) in occipital electrodes (n = 40). The median split was performed based on ACW-e^−1^, and variables that had shown statistically significant results in the mediation model (*power*, *power ratio* and CRS-R) were separated according to the split. Independent t tests revealed that subjects in the top ACW quantile (*power*: M = −3.48, SD = 0.23), displayed lower *power* than subjects in the bottom quantile (M = −3.18 m SD = 0.26), *t*(38) = 3.89, p < 0.001. Similarly, for *power ratio*: top quantile (M = 0.66, STD = 0.064) and bottom quantile (M = 0.82, STD = 0.092) displayed statistically significant differences, with the top quantile having a lower *power ratio*, *t*(38) = 3.89, p < 0.001. CRS-R also exhibited significant differences between the top quantile (median = 5) and the bottom quantile (median = 7), Mann-Whitney *U* = 284, p = 0.022. In contrast to median splits based on ACW, no statistically significant differences for CRS-R were found when attempting splits based on *power* or *power ratio* (Fig. 7B). This suggests a special role of ACW in sorting and distinguishing individual DOC patients with respect to their state of consciousness.

Taken together, we show that DOC patients who exhibit a spectral peak (either in the alpha or theta frequency ranges) tend to have a shorter ACW. Secondly, DOC subjects with a prolonged ACW less commonly exhibit spectral peaks. Thirdly, DOC individuals with longer ACW-e^−1^ tend to have lower CRS-R scores. In summary, we provide compelling evidence for a link between ACW, spectral peaks and the state of consciousness, with ACW potentially serving as a central component in this intricate relationship**.**

### 3.6 Classifying the state of consciousness with spectral measures and ACW

Next, we aimed at exploring whether the conjoint use of ACW and spectral measures would allow the classification of subjects based on their state of consciousness. Employing an SVM model, our objective was to classify subjects into two categories initially (controls and DOCs), followed by three categories (controls, MCS and UWS). For this analysis, we aggregated data obtained from both frontal and occipital channels. Only datapoints related to alpha peaks were utilized, given that no healthy subjects presented theta peaks. Consequently, the dataset comprised 44 healthy controls (22 frontal and 22 occipital) and 20 DOC subjects (7 frontal and 13 occipital). Three models were employed for each classification task: one utilizing only alpha peak features, a second using solely ACW features and finally, one combining both alpha and ACW features.

For our initial classification task (Controls vs. DOC), all models demonstrated high accuracy (see Supplementary Table 1). Specifically, the alpha peak-only model achieved 93.57 % (± 8.33 %) accuracy, the ACW-only model achieved 90.48 % (± 11.39 %) accuracy, while the combined alpha peak and ACW model attained the highest accuracy of the three (95.48 ± 7.31 %). Similarly, precision, recall, F1 score, and AUC yielded very high values. For our second classification task (Controls vs. MCS vs. UWS), accuracy witnessed a notable drop across all three models: the alpha peak-only model achieved 77.62 % (± 9.47 %) accuracy, the ACW-only model attained the highest accuracy (79.52 % ± 13.05 %), while the conjoint model achieved 78.33 % ± 12.57 % accuracy. Overall, precision, recall, F1 score, and AUC remained high, ranging from 90 to 95 % (see Supplementary Table 2 for complete results). It is worth noting, though, that these metrics are largely influenced by the number of true positives. Indeed, as depicted in Supplementary Fig. 5, the combined alpha peak-ACW model achieved nearly perfect classification of healthy subjects into their respective category (misclassifying only 3 out of 44 healthy individuals out as MCS, and thus resulting in a high true positive rate). However, the model encountered difficulty in distinguishing between MCS and UWS, correctly classifying only 6 out of 15 MCS and 3 out 5 UWS subjects, leading to higher misclassification rates.

In summary, our machine learning analysis demonstrates that alpha peak spectral measures and ACW are effective in discriminating between healthy controls and DOC individuals. However, their utility in distinguishing between MCS and UWS remains limited.

## 4 Discussion

In the present study, we tackle one of the most consistent observations in the resting state EEG of patients with disorders of consciousness: the decrease in power in the alpha range compared to healthy controls. We approach this finding as a weakening or disappearance of the alpha peak and exploit it for the clinical diagnosis of the state of consciousness in DOC subjects. We perform a peak analysis on the PSD and measure INTs (probed through ACW) from EEG signals obtained from twenty-five healthy controls and eight-eight DOC patients.

### 4.1 Quantifying the weakening of the alpha peak in DOC

A decrease in resting-state power in the alpha band consistent with a decrease in the state of consciousness has already been reported (Engemann et al., 2018, Lechinger et al., 2013, Rossi Sebastiano et al., 2015, Sitt et al., 2014). We extended this finding by employing peak-analysis aimed at specifically quantifying not only the decline in power, but also the weakening of the alpha peak in its characteristics (i.e. peak-related measures). Alpha peak measures show significant decreases in DOC individuals across almost all measures in both frontal and occipital electrodes, with the only exception being *maximum power* (which shows an increase in DOC subjects) and *width*. Additionally, we note the shifting of the alpha peak to the theta band in several DOC individuals. This phenomenon has been reported in MCS subjects (Schiff et al., 2014) and has also been observed in healthy subjects under the influence of ketamine (Colombo et al., 2019, Waschke et al., 2021, Zilio et al., 2023). Importantly, patients administered with ketamine report dream-like experiences upon emergence from anesthesia (Sarasso et al., 2015) indicating the presence of consciousness and opening up the question of whether patients in MCS could share a similar experience. Further research should aim to replicate these findings and explore whether a peak in power in the theta band could potentially serve as an indirect correlate of a reduced/altered, yet present, state of consciousness.

Regarding *maximum power*, multiple studies have linked high amplitude of delta waves to states of reduced or loss of consciousness, such as deep sleep (Brown et al., 2010, Murphy et al., 2011, Zilio et al., 2021) and anesthesia (Murphy et al., 2011, Purdon et al., 2013, Zilio et al., 2021). Further, an increase in delta power has been demonstrated as a consistent finding in patients with disorders of consciousness (Chennu et al., 2014, Engemann et al., 2018, Lechinger et al., 2013, Rossi Sebastiano et al., 2015, Sitt et al., 2014, Zilio et al., 2021). While “*maximum power*” in our measurement of alpha or theta peak does not necessarily correspond to the maximum power in the delta band itself, it is worth noting that the PSD for EEG signals generally exhibits an inverse exponential trend, placing *maximum power* almost invariably in the delta range. With this limitation in mind and considering the different methodology employed in our study, we indirectly replicate the finding of increased delta power in DOC patients.

### 4.2 Prolongation of INTs mediates the impact of theta peak on the level of consciousness

First, we replicate the finding of prolonged INTs in DOC patients with respect to control individuals (Buccellato et al., 2023, Murphy et al., 2011, Zilio et al., 2021). Our study replicates and extends previous results through the inclusion of ACW-e^−1^ and adds to the growing body of literature on INTs and their relation to consciousness, neurological and mental disorders (Golesorkhi et al., 2021b, Northoff et al., 2021, Uscătescu et al., 2021, Watanabe et al., 2019, Xu et al., 2023, Zhang et al., 2023).

The negative correlations between our peak measures and ACW indicate that the weakening of both alpha and theta peak is strongly related to prolongation of the INTs. Moreover, controls and patients with absence of a spectral peak exhibit a longer ACW (see Supplementary Figs. 2 and 3), which further extends the relation between spectral peaks and INTs along the continuum from the weakening of alpha and theta peaks to their disappearance. These results appear robust, with correlations applying both to controls and DOC patients.

In our study, we find weak-to-moderate correlations between ACW and CRS-R in occipital electrodes for subjects displaying a theta peak (n = 27). The mediation analysis pointed at the possible explanation of INTs as mediator in the relationship between theta peaks and the level of consciousness (i.e. CRS-R).

On a related note, previous research has found that some healthy subjects may demonstrate low amplitude or no alpha peak, a phenotype known as Low-Voltage EEG (LVEEG) (Bazanova and Vernon, 2014). LVEEG has an elusive definition, with some studies defining it as a low voltage across the whole frequency spectrum (Synek, 1983) and others restricting their definition specifically to the alpha band (Anokhin et al., 1992). Bazanova and colleagues (Bazanova and Vernon, 2014) report a prevalence ranging between 3 and 13 % in healthy adults, in line with our findings (i.e. 12 %). Given our correlational findings, we suggest, albeit tentatively, that LVEEG may be related to longer INTs in these healthy subjects. However, this remains to be further tested in the future.

### 4.3 Peak analysis and INTs as potential markers for the state of consciousness

The correlations observed between ACW and CRS-R, coupled with our mediation analysis findings, suggest the possibility of utilizing our spectral measures and ACW as markers for the state of consciousness. This possibility was first explored through split analyses, demonstrating that the conjoined ACW and peak measures yielded good discrimination between controls and DOC. This, in turn, guided our machine learning analyses, which demonstrated high accuracy (95.5 %) for the combined alpha peak-ACW model in discriminating controls from DOC subjects, indicating that the weakening, shifting to the theta range and, ultimately, the disappearance of the alpha peak might possess important information for assessing the state of consciousness.

While further research is needed, the use of peak analysis and ACW could potentially serve as a relatively straightforward method for clinicians to obtain a preliminary assessment of a patient’s consciousness state through a quick glance at a PSD or at an Autocorrelation Function (ACF) graph. Indeed, the pronounced differences in our measures between controls and DOC individuals are readily apparent upon visual inspection (e.g., see Fig. 2 for a comparison of PSDs between healthy controls and DOC patients, and Fig. 4A for two ACF graphs depicting healthy and DOC individuals). However, our machine learning results suggest that our measures currently lack the precision required for a more fine-grained clinical distinction of the state of consciousness, such as discriminating between MCS and UWS.

However, as demonstrated in previous machine learning studies (Engemann et al., 2018, Sitt et al., 2014), optimal outcomes in classifying subjects based on their state of consciousness are achieved when combining EEG markers of diverse natures: each marker serving a unique role, with some excelling in discriminating controls and UWS, while others in distinguishing between MCS and UWS, and so forth. For instance, ACW and Peak analysis measures, by effectively differentiating healthy individuals from DOC, may aid in bridging the gap between behavior and consciousness by identifying cases of Cognitive Motor Dissociation (CMD) (Schiff, 2015, Schnakers et al., 2022, Young et al., 2024). Further validation is needed to determine whether more fine-grained measures of ACW and spectral peak analysis could allow for an accurate distinction between MCS and UWS.

### 4.4 INTs and Alpha peak through the lens of the Temporo-spatial Theory of Consciousness (TTC)

The Temporo-spatial Theory of Consciousness (TTC) has recently emerged as a promising alternative to more established theories of consciousness (Northoff and Huang, 2017, Northoff and Lamme, 2020, Northoff and Zilio, 2022). TTC places particular emphasis on the brain’s spontaneous activity and its interaction with post-stimulus activity. Central to the theory are the brain’s temporal dynamic and spatial topography with both converging to produce conscious experience.

Within this framework, INTs are posited as a key mechanism for consciousness due to their crucial role in processing inputs. TTC suggests that INTs are responsible for the brain’s “temporo-spatial alignment,” solving the long-standing distinction between figure and background in conscious perception. According to TTC, INTs represent the form or structure of consciousness – the context or “set” where the conscious scene unfolds, serving as the background to conscious content. Conversely, TTC identifies the brain’s pre-stimulus activity, with special focus on the alpha band, as key for conscious content (as opposed to the background previously described).

The relationship between alpha peak and INTs, which could be viewed through the TTC framework as the relationship between figure and background has not been thoroughly explored. However, Buccellato et al., 2023 identified a negative correlation between alpha peak frequency (APF), measured via frequency sliding, and ACW. Interestingly, this correlation breaks down in states of reduced consciousness, such as under anesthesia and, importantly, in DOC.

Our study extends these findings to different alpha characteristics. We note that correlations between alpha and ACW persist in DOC patients, contrasting with the disruption seen by Buccellato and colleagues. However, what we find most intriguing is the potential relationship between peak measures and CRS-R, with INTs acting as a mediator, as hinted by our mediation analysis (albeit conducted on theta peaks). From a strict TTC perspective, these mediation results are not entirely unexpected. According to TTC, INTs reflect the neural predisposition of consciousness: while they may not directly give rise to conscious content, they could hold a fundamental role in allowing its formation, giving it a structure and an outline. Just as a figure cannot exist without a background, conscious content requires the “set” provided by INTs to emerge.

However, it is important to note that properly addressing TTC’s predictions about the relationship between figure and background would require a task paradigm to specifically analyze the interaction between pre- and post-stimulus activity.

### 4.5 Limitations

Important limitations regarded the sample size, which for most of our analyses was low (especially for mediation analysis, which requires longitudinal data and large sample sizes to obtain reliable results) (Fairchild and McDaniel, 2017), a direct consequence of the fact that most DOC patients did not show any spectral peaks in the frequency bands of interest. Indeed, our approach through peak analysis here exhibits its greatest weakness, that is, not being applicable to all participants. We also remind the reader that seven DOC patients shifted from one diagnostic category to another during the follow-up period, which could potentially compromise subject weighting.

Additionally, multiple factors are known to influence alpha band amplitude, such as, for example, cerebral blood flow (with power increasing as blood flow increases) (O’Gorman et al., 2013). Importantly, suppression of the alpha band waves is obtained upon eye-opening, a phenomenon known as the Berger effect (Kirschfeld, 2005). Indeed, these possible confounding factors were not accounted for in our research, with half of our patients being affected by stroke (and therefore likely having significantly impaired or altered cerebral blood flow) and whether DOC patients had their eyes opened or closed at the moment of recording was not documented.

Furthermore, research on quantitative EEG undermines the idea of alpha power being specifically linked to the state of consciousness. Some studies have found a decreased relative alpha power in subjects with mild traumatic brain injury (the second most common cause of DOC in our dataset) (Lewine et al., 2019), while a lower relative alpha power appears to be a key prognostic marker in stroke patients (Bentes et al., 2018) (stroke being the leading cause of DOC in our dataset). A recent study conducted by Colombo and colleagues (Colombo et al., 2023) has found that a decrease in alpha power could discriminate exclusively between anoxia and non-anoxia DOC individuals, but not between conscious and unconscious subjects. The authors concluded that alpha power suppression might indicate widespread cortical damage, rather than carrying specific information about the state of consciousness. Indeed, the studies referenced in this paragraph, along with recent research (Maschke et al., 2024), highlight the crucial role of etiology in DOC, identifying it as a major confounding factor in the search for markers for consciousness.

Finally, our study, like many others investigating markers for the state of consciousness, measures the validity of its proposed markers using the CRS-R as benchmark – the same scale that researchers seek to overcome. This presents a complex epistemological challenge (Neveu et al., 2023) that we acknowledge, although we do not intend to tackle.

## 5 Conclusion

Building upon existing literature, we emphasize the connection between the decrease in power within the alpha band and the level or state of consciousness — a relationship that remains to be fully understood. Through peak analysis, we quantified and measured the alpha peak itself, showing its weakening, shift to theta peak, and/or complete disappearance in the resting state EEG of patients with DOC. This was accompanied by prolongation of INTs, as measured through ACW, which correlates with standardized behavioral assessment of consciousness (i.e. CRS-R) in DOC subjects. Mediation analysis hinted at the possibility of ACW mediating the relationship of alpha/theta peak with the level of consciousness. Finally, through data sorting with split analyses and machine learning, we demonstrate that alpha peak and INTs conjointly can effectively differentiate controls and DOC individuals with high accuracy. In conclusion, we highlight the intricate relationship between alpha/theta peaks, ACW and the state of consciousness (i.e. CRS-R) which, tentatively, lays the groundwork for future research aimed at exploring these measures as potential clinical biomarkers of the level or state of consciousness.

## Data and code availability statements

The data used in this article is sensitive and abides by specific privacy regulations. Custom scripts used in this study are available upon reasonable request. Relevant code to replicate our analysis is available at https://www.georgnorthoff.com/code.

## CRediT authorship contribution statement

**Ezequiel Pablo Espinosa:** Writing – review & editing, Writing – original draft, Visualization, Software, Investigation. **Di Zang:** Writing – review & editing, Investigation, Funding acquisition. **Andrea Buccellato:** Writing – review & editing, Investigation, Data curation. **Zengxin Qi:** Investigation. **Xuehai Wu:** Investigation. **Samira Abbasi:** Formal analysis. **Yasir Catal:** Writing – review & editing, Software. **Stephan Lechner:** Writing – review & editing, Software. **Federico Zilio:** Writing – review & editing, Conceptualization. **Georg Northoff:** Writing – review & editing, Supervision, Project administration, Funding acquisition, Conceptualization.

## Funding

This research has received funding from the European Union’s Horizon 2020 Framework Program for Research and Innovation under the Specific Grant Agreement no, 785907 (Human Brain Project SGA2). G.N. is grateful for funding provided by UMRF, uOBMRI, CIHR and PSI. We are also grateful to CIHR, NSERC, and SSHRC for supporting our tri-council grant from the Canada–UK Artificial Intelligence (AI) Initiative The self as agent–environment nexus: crossing disciplinary boundaries to help human selves and anticipate artificial selves’ (ES/T01279X/1) (together with Karl J. Friston from the UK).

This work was also supported by the National Natural Science Foundation of China (Nos. 82271224); Shanghai Municipal Science and Technology Major Project (No.2018SHZDZX01), ZJ Lab, and Shanghai Center for Brain Science and Brain-Inspired Technology; SHANGHAI ZHOU LIANGFU MEDICAL DEVELOPMENT FOUNDATION “Brain Science and Brain Diseases Youth Innovation Program”; National High Level Hospital Clinical Research Funding (No. 2023-NHLHCRF-BQ-43).

## Declaration of Competing Interest

The authors declare that they have no known competing financial interests or personal relationships that could have appeared to influence the work reported in this paper.

## Data Availability

The data that has been used is confidential.
