# Supplementary material for: Spectral peak analysis and intrinsic neural timescales as markers for the state of consciousness
Source: Neuroimage Clin. 2024 Oct 30;44:103698. doi: 10.1016/j.nicl.2024.103698 (PMC11574811; doi:10.1016/j.nicl.2024.103698)
Supplement: Supplementary Data 1 [file mmc1.docx]

## **Supplementary figures and tables**


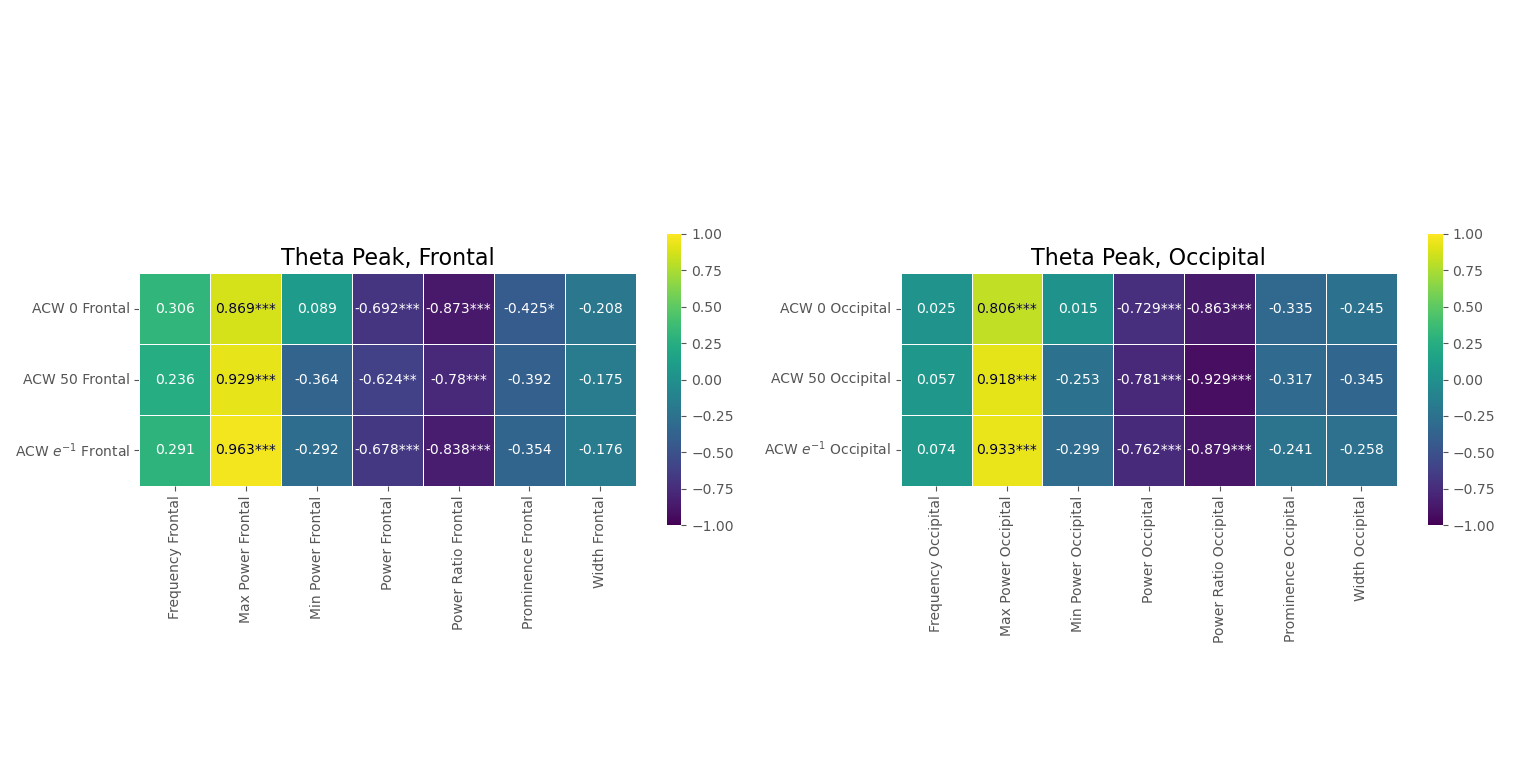


**Supplementary figure 1 – Correlations between ACW and alpha spectral measures.**

Correlations between ACW and alpha spectral measures (both in controls and in DOC) are present in theta peak DOC individuals too.


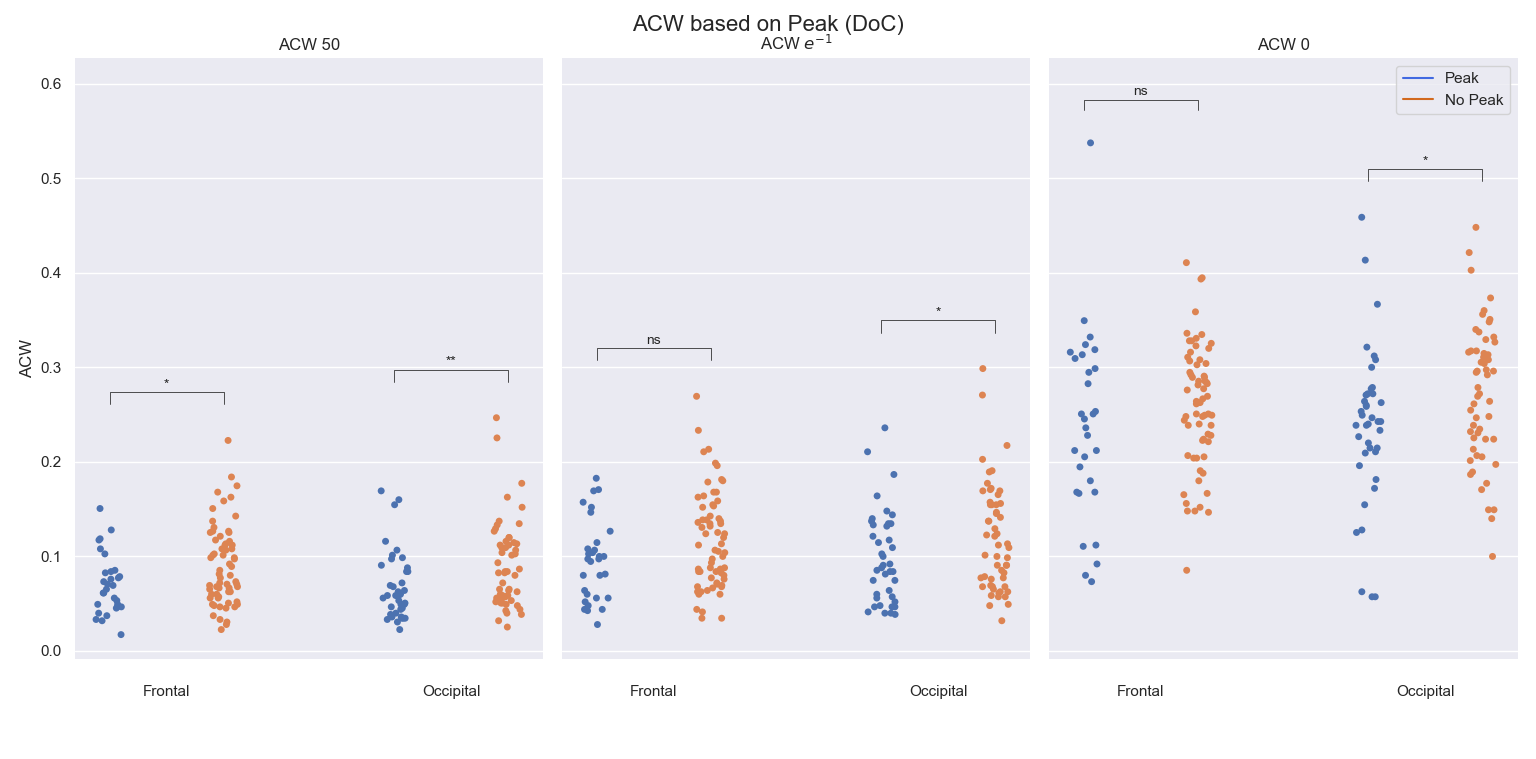


**Supplementary figure 2 – ACW in patients with and without a peak.**

DOC patients with a peak appear to have a shorter ACW, on average. However, only occipital electrodes reveal statistically significant differences.


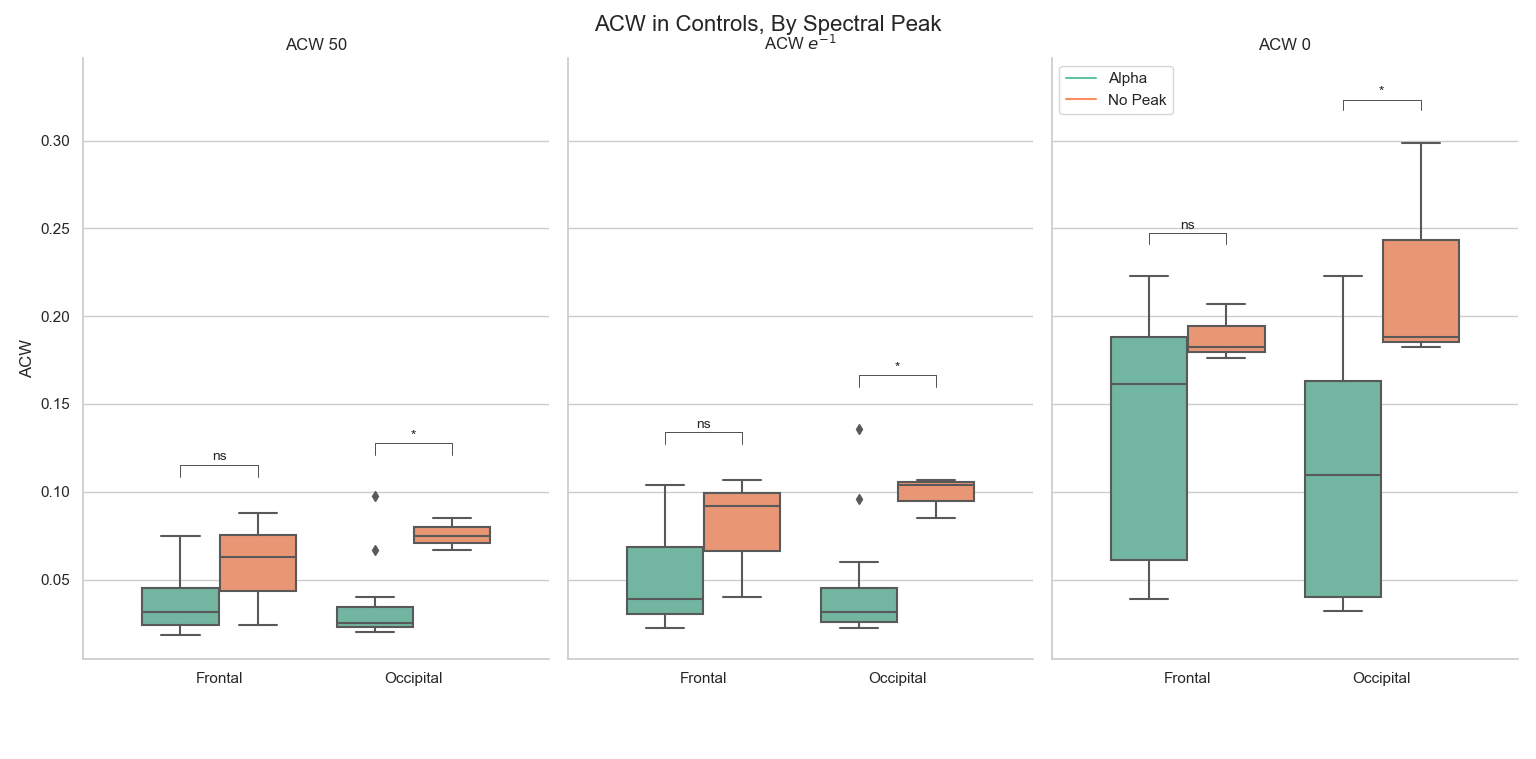


**Supplementary figure 3 – ACW in healthy controls with and without a peak.**

Similarly to DOC patients, controls without a peak appear to exhibit a longer ACW. Given the scarce number of subjects without a peak in the control group (frontal = 3, occipital = 3) results should be interpreted with caution.


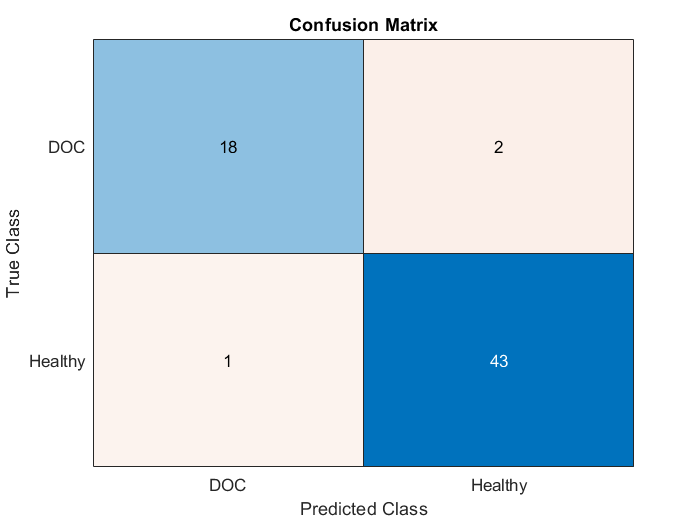


**Supplementary figure 4 – Confusion Matrix for the combined alpha peak-ACW SVM model, classifying Healthy Controls vs. DOC subjects**.


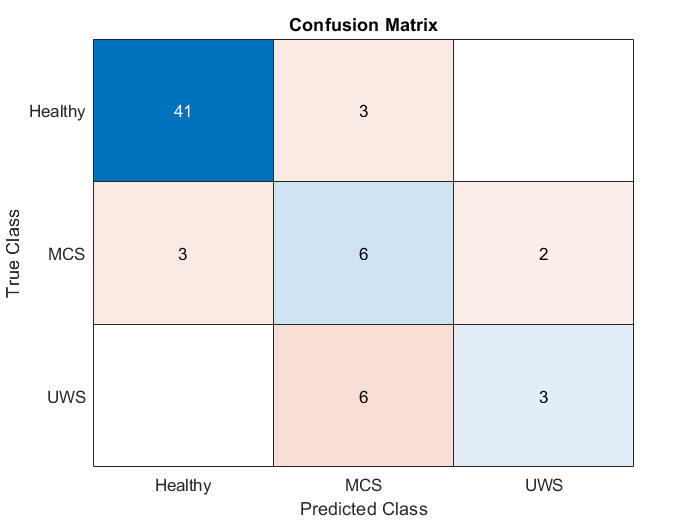


**Supplementary figure 5 – Confusion matrix for healthy-MCS-UWS classification problem.**

The alpha peak-ACW SVM model correctly identifies healthy controls with extremely high accuracy. However, spectral measures and ACW do not appear to hold relevant information for distinguishing between MCS and UWS.

**Supplementary table 1 – Results for healthy-DOC classification problem across 10 folds.**

|  | **Accuracy** | **Precision** | **Recall** | **F1 Score** | **AUC** |
| --- | --- | --- | --- | --- | --- |
| **Alpha Features only,** | 93.57% (±8.33%). | 90.50% (±12.35%) | 100% (±0%). | 94.60% (±7.03%) | 86.67% (±31.23%) |
| **ACW Features only** | 90.48% (±11.39%) | 88.33% (±17.66%) | 98.00 (±6.32%) | 91.74% (±11.14%) | 82.75% (±34.61%) |
| **Alpha and ACW Features,** | 95.48% (±7.31%) | 95.50% (±9.56%) | 98.57% (±4.52%) | 96.69% (±5.55%) | 88.89% (±31.43%) |

ACW: autocorrelation window.

**Supplementary table 2 – Results for healthy-MCS-UWS classification problem across 10 folds.**

|  | **Accuracy** | **Precision** | **Recall** | **F1 Score** | **AUC** |
| --- | --- | --- | --- | --- | --- |
| **Alpha Features only,** | 77.62% (±9.47%). | 92.33% (±9.94%) | 100% (±0%). | 95.76% (±5.51%) | 90.00% (±31.62%) |
| **ACW Features only** | 79.52% (±13.05%) | 89.00% (±12.38%) | 98.00 (±6.32%) | 92.87% (±8.31%) | 86.50% (±31.45%) |
| **Alpha and ACW Features,** | 78.33% (±12.57%) | 94.33% (±9.17%) | 94.00% (±13.50%) | 93.26% (±8.32%) | 90.00% (±31.62%) |

ACW: autocorrelation window.
